# Paraganglioma and pheochromocytoma upon maternal transmission of *SDHD* mutations

**DOI:** 10.1186/s12881-014-0111-8

**Published:** 2014-10-10

**Authors:** Jean-Pierre Bayley, Rogier A Oldenburg, Jennifer Nuk, Attje S Hoekstra, Conny A van der Meer, Esther Korpershoek, Barbara McGillivray, Eleonora PM Corssmit, Winand NM Dinjens, Ronald R de Krijger, Peter Devilee, Jeroen C Jansen, Frederik J Hes

**Affiliations:** Department of Human Genetics, Leiden University Medical Center, PZ S-04, P.O. Box 9600, 2300 RC Leiden, the Netherlands; Department of Clinical Genetics, Leiden University Medical Center, Leiden, the Netherlands; Department of Pathology, Leiden University Medical Center, Leiden, the Netherlands; Department of Otorhinolaryngology, Leiden University Medical Center, Leiden, the Netherlands; Department of Endocrinology, Leiden University Medical Center, Leiden, the Netherlands; Department of Clinical Genetics, Erasmus Medical Center Rotterdam, Rotterdam, the Netherlands; Department of Pathology, Josephine Nefkens Institute, Erasmus Medical Center Rotterdam, Rotterdam, the Netherlands; Hereditary Cancer Program, BC Cancer Agency, Vancouver, Canada

**Keywords:** Paraganglioma, Pheochromocytoma, SDHD, Imprinting, Parent-of-origin

## Abstract

**Background:**

The *SDHD* gene encodes a subunit of the mitochondrial tricarboxylic acid cycle enzyme and tumor suppressor, succinate dehydrogenase. Mutations in this gene show a remarkable pattern of parent-of-origin related tumorigenesis, with almost all *SDHD*-related cases of head and neck paragangliomas and pheochromocytomas attributable to paternally-transmitted mutations.

**Methods:**

Here we explore the underlying molecular basis of three cases of paraganglioma or pheochromocytoma that came to our attention due to apparent maternal transmission of an *SDHD* mutation. We used DNA analysis of family members to establish the mode of inheritance of each mutation. Genetic and immunohistochemical studies of available tumors were then carried out to confirm *SDHD*-related tumorigenesis.

**Results:**

We found convincing genetic and immunohistochemical evidence for the maternally-related occurrence of a case of pheochromocytoma, and suggestive evidence in a case of jugular paraganglioma. The third case appears to be a phenocopy, a sporadic paraganglioma in an *SDHD* mutation carrier with no immunohistochemical or DNA evidence to support a causal link between the mutation and the tumor. Microsatellite analysis in the tumor of patient 1 provided evidence for somatic recombination and loss of the paternal region of chromosome 11 including *SDHD* and the maternal chromosome including the centromere and the p arm.

**Conclusions:**

Transmission of *SDHD* mutations via the maternal line can, in rare cases, result in tumorigenesis. Despite this finding, the overwhelming majority of carriers of maternally-transmitted mutations will remain tumor-free throughout life.

## Background

Paragangliomas of the head and neck (HNPGL) are rare and often benign tumors that arise most commonly in the carotid body, but also occur in the jugular bulb or tympanic nerve and at the vagal bodies of the ganglions of the vagal nerve [1]. HNPGLs are generally diagnosed in adulthood and show mild symptoms, with a characteristically slow tumor progression [2]. Pheochromocytomas and abdominal paragangliomas are closely related tumors that are associated with the sympathetic nervous system. They occur most commonly in the adrenal medulla (as pheochromocytomas) but approximately 10-20% arise elsewhere in the abdomen [3]. These non-adrenal tumors are collectively referred to as ‘sympathetic paragangliomas’ (sPGLs).

Succinate dehydrogenase (SDH) is a tetrameric mitochondrial enzyme that consists of two catalytic subunits, SDHA and SDHB, and two membrane-anchoring subunits, SDHC and SDHD. SDH plays a central role in the tricarboxylic acid cycle and the electron transport chain, the two essential energy producing processes of the cell. The last decade has seen the identification of mutations in five SDH-related genes that cause hereditary paraganglioma-pheochromocytoma syndrome, including *SDHD* (chromosome 11q23) [4], *SDHB* (chromosome 1p36) [5], *SDHC* (chromosome 1q23) [6], *SDHAF2* (chromosome 11q12.2) [7], and *SDHA* (chromosome 5p15) [8].

Germline mutations of the *SDHD* gene show a ‘parent-of-origin’ expression phenotype, with tumor development occurring only when mutations are inherited via the paternal line. This phenotype was originally interpreted as evidence for ‘imprinted’ or allele-specific gene expression of *SDHD* [9]. This phenomenon is not seen in the case of *SDHA*, *SDHB* or *SDHC* gene mutations, which result in tumor development regardless of the parental origin of the mutation. The only other tumor suppressor genes known to show a ‘parent-of-origin’ phenotype are the recently described genes *SDHAF2* and *MAX,* located on chromosome 11q12.2 and 14q23, respectively [7,8,10].

Previous cases of tumor development related to maternal transmission of an *SDHD* mutation include a 2008 report by Pigny et al. [11], which was later challenged as a probable misdiagnosis [12], and more recent report by Yeap et al. in which the authors presented genetic evidence of maternal transmission [13].

Here we describe a patient with pheochromocytoma and two patients with head and neck paraganglioma who came to our attention due to tumor susceptibility that was apparently maternally-related. We first investigated available family members to exclude the possibility that the mutation could have been transmitted by the biological father. We then analysed the loss of alleles of the *SDHD* gene in available tumors, we carried out whole chromosome loss of heterozygosity (LOH) analysis and finally, we analysed the expression of SDHB in tumors, loss of which is a hallmark of tumors related to succinate dehydrogenase dysfunction.

## Methods

### Patients

The patients described in this study carried a confirmed pathogenic mutation in the *SDHD* gene, identified due to a clinical diagnosis or to a family relationship to known mutation carriers. Patients and family members were seen at the relevant centers in Leiden or Rotterdam, the Netherlands, or in Vancouver, Canada. Patients and other currently unaffected family members known to be mutation carriers underwent a full clinical assessment. Written informed consent was obtained for DNA testing, further analyses and publication of all results, according to protocols approved by the Ethics Committees of the Erasmus Medical Center, Rotterdam and the BC Cancer Agency, Vancouver. Verbal informed consent was obtained from patients seen at the LUMC, Leiden.

### Haplotype analysis

DNA was isolated from heparinized whole blood according to standard protocols. Analysis of haplotypes using polymorphic di-, and tetra nucleotide markers (microsatellite markers) was performed following standard procedures (details available upon request), using the markers described in the results section. These markers were selected based on location, and for probable informativity due to a high reported heterozygosity index, from a custom database of 8100 markers based on the UniSTS and Marshfield databases (available upon request).

### Allele specific loss of *SDHD*

The loss of a specific parental allele of *SDHD* was determined by PCR and bi-directional Sanger sequencing of the *SDHD* gene using standard procedures (primer details available upon request).

### Loss of heterozygosity (LOH) analysis

Tumor sections (7um) were incubated overnight with proteinase K at 60°C and DNA was isolated using the Qiagen FFPE DNA kit (Qiagen Benelux B.V., Venlo, The Netherlands) according to the manufacturer’s instructions. A selection of informative microsatellite markers were analysed, as described in the results section. Paragangliomas often remain histologically well-differentiated and contain several types of normal cells that show expansion under the influence of the chief cell fraction, the only neoplastic component of the tumor [14]. The presence and expansion of these bystander cells means that loss of heterozygosity analysis is often contaminated by the presence of DNA from these cell populations. We therefore used the allelic imbalance ration of 0.7 or less as evidence for LOH [15]. Microsatellite markers were analyzed on an ABI 3730 genetic analyzer and using Gene Marker software (Soft Genetics, State College, PA 16803, USA), with ABI GeneScan Rox 400 as internal size standards. LOH of markers was calculated using the allelic imbalance ratio: AIR = (Tumor1/Tumor2)/(Normal1/Normal2). Ratios were based on results from the duplo analysis of two separate DNA isolations from the tumor. Some markers were either not informative in the patient or did not perform well enough with tumor DNA to give a reliable result. The remaining informative markers (n = 7) were used in the analysis.

### Pathology and (immuno)histochemistry

Sections from formalin-fixed paraffin embedded (FFPE) tumor blocks were stained with hematoxylin & eosin (H&E) using standard methods. An experienced neuroendocrine pathologist (RdK) evaluated slides and provided or confirmed (patient 3) the histopathological diagnosis. A recent and important development in the diagnosis of SDH-related paraganglioma has been the application of SDHB immunohistochemistry, which can reliably identify and differentiate SDH-related tumors from tumors with other causes regardless of which of the SDH genes is actually affected by a mutation [16]. SDHB immunohistochemistry was carried out as described [16], using a rabbit polyclonal primary SDHB antibody, HPA002868 (Sigma-Aldrich Corp; St Louis, MO, USA; 1:500).

## Results and discussion

**Figure 1:**
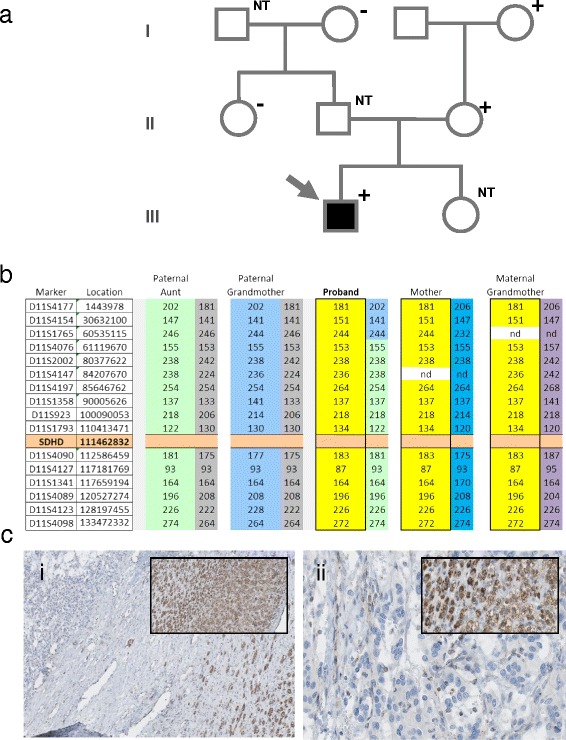
**Patient 1 – pedigree and immunohistochemistry. a)** An arrow indicates the proband. Filled boxes indicate paraganglioma and plus or minus the *SDHD* mutation status. NT = not tested. **b)** Chromosome 11 haplotypes of family members. Microsatellite markers are shown with genomic location (marker D11S4177 is close to the telomere of the p arm. Marker D11S4098 is close to the telomere of the q arm), and the position of *SDHD* is indicated. Alleles in bright yellow blocks represent the probable disease haplotype, present in the proband, mother and maternal grandmother. Other colors represent probable additional haplotypes in the family and possible recombinations; nd = not determined. **ci)** SDHB immunohistochemistry of the pheochromocytoma, 25×. Inset, adrenal cortex positive for SDHB, 25×. **cii)** Detail of adrenal medulla, 200×, with inset of positive cortex, 200×.

Our first patient (patient 1) was a young man who experienced increasing tiredness around the age of 16, but without excessive transpiration or palpitations. The patient also reported sudden weight gain and acute, localized headaches. The evaluation of catecholamines showed raised plasma concentrations of noradrenaline, adrenaline, dopamine and raised urinary levels of normetanephrine. Radiological examination (by CT) revealed a mass at the level of the left kidney, and a positive octreotide scan showed a suspicious accumulation at the left adrenal. A tumor (70 × 60 × 40mm) was removed via adrenalectomy and was confirmed as a pheochromocytoma with signs of vasoinvasive growth. Although distant maternal relatives of this patient have a history of paragangliomas and carry a known pathogenic *SDHD* gene mutation, no immediate maternal relative is affected, probably due to the inheritance of the mutation via the maternal line for several generations. The paternal family had no known history of paraganglioma. Due to the maternal inheritance of the mutation, the patient was not immediately suspect for a *SDHD*-related paraganglioma. The initial mutation analysis therefore focused on other genes including *VHL* (with FISH analysis), *RET*, *SDHB* and *SDHC*. When all proved to be negative, analysis of the *SDHD* gene was undertaken and a heterozygous mutation identified in exon 3, the well-known Dutch founder mutation, c.274G>T, p.Asp92Tyr, also present in other family members. The patient, his mother and his maternal grandmother all carried the mutation (Figure 1a).

Analysis of microsatellite markers in patient 1 and four other family members (Figure 1b) indicated that the patient indeed inherited a chromosome-wide, mutation-associated haplotype from his mother, via the maternal grandmother. The paternal chromosome, also represented in the paternal grandmother and paternal aunt, was not associated with a mutation of *SDHD*, indicating that the patient inherited the mutation via the maternal line.

Review of H&E tumor sections from the pheochromocytoma showed a typical morphology (Figure 1ci) and anti-SDHB immunohistochemistry revealed specific loss of SDHB protein expression in the adrenal medulla (Figure 1cii).

**Figure 2:**
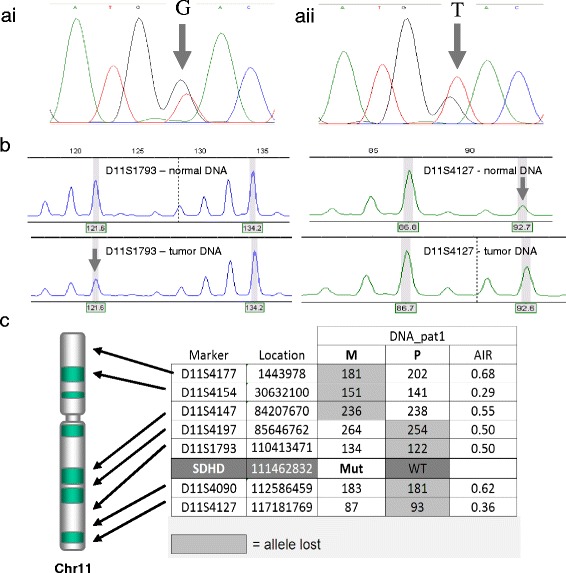
**Patient 1 – tumor analysis. a)** Sanger sequencing of *SDHD* in normal (ai) and tumor (aii) DNA. Arrows indicate the relevant nucleotides in the heterozygous patient. **b)** Typical profiles of microsatellite marker alleles showing loss of heterozygosity. Arrows indicate the allele lost. **c)** Table indicating loss of microsatellite marker alleles and parental origin, with approximate positions indicated on an ideogram of chromosome 11.

Microdissection of tissue from the adrenal medulla, followed by PCR analysis and sequencing of *SDHD*, showed that the wild type allele (guanine (G) nucleotide – arrow, Figure 2ai) is prominent in the DNA from blood of patient 1 but is underrepresented in tumor DNA (Figure 2aii), indicating loss of heterozygosity in the tumor. While not showing complete loss, this result is typical of LOH in paraganglioma which show complex and significant admixture of normal cells, largely maintaining their normal tissue architecture and cellular composition of normal cell types, which also proliferate together with tumor cells.

As we wished to evaluate the extent and parental origin of chromosomal loss, tumor DNA was analyzed for loss of heterozygosity using a selection of polymorphic microsatellite markers. Tumor DNA from patient 1 showed chromosome-wide loss of heterozygosity, with allelic imbalance ratios (AIR) of <0.7. Interestingly, analysis of the parental origin of the alleles showed only partial loss of the paternal chromosome, with loss of maternal alleles on the p arm and centromeric q arm of chromosome 11 (Figure 2ci). This result is most readily explained by somatic recombination in a tumor progenitor cell, followed by loss of a composite chromosome consisting of the paternal (unmutated) allele of SDHD and the maternal p arm of chromosome 11.

**Figure 3:**
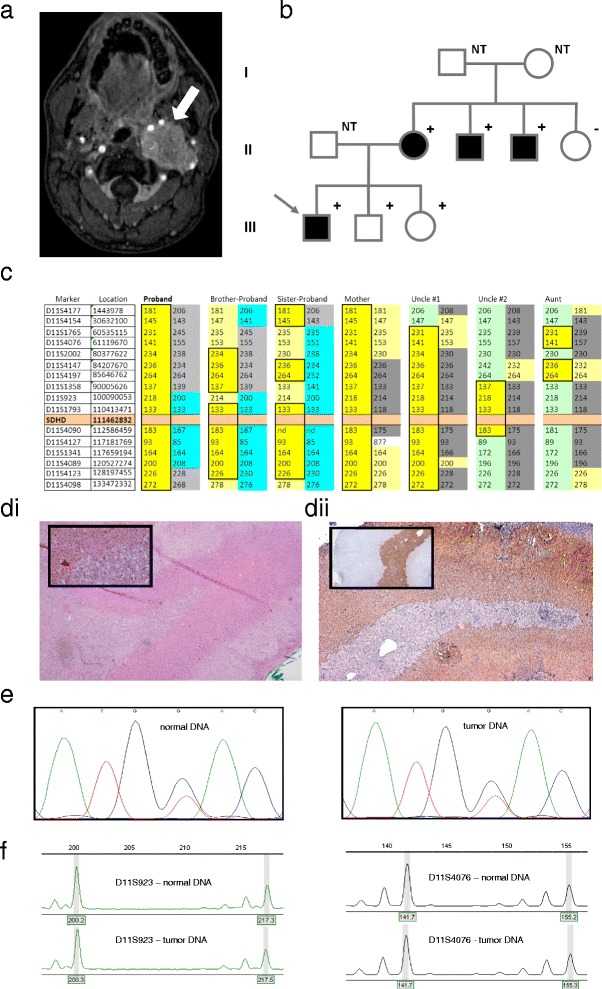
**Patient 2 – pedigree, DNA and tumor analysis. a)** MRI of the skull base in patient 2, showing a probable vagal body tumor (white arrow). **b)** Pedigree of family 2 - arrow indicates the proband, filled boxes indicate paraganglioma, and plus or minus the *SDHD* mutation status. NT = not tested. **c)** Chromosome 11 haplotypes of family members. Alleles in bright yellow blocks represent the likely disease haplotype, present in all mutation carriers. Other colors represent probable additional haplotypes in the family and possible recombinations. The proband and siblings all carry the disease haplotype. **d)** Histochemistry and immunohistochemistry: d1) HE staining of adrenal tumor, 25×, with inset 200×. d2) SDHB immunohistochemistry of adrenal tumor, 25×. Inset, chromogranin A staining, 25×. **e)** Sanger sequencing of *SDHD* in normal and tumor DNA. **f)** Typical profiles of microsatellite marker alleles showing no loss of heterozygosity.

The second patient was also a young man, aged 17 at the time of diagnosis of bilateral paragangliomas. The tumors were diagnosed using MRI and consisted of a left-sided vagal tumor (30 × 40 × 45mm) encasing the carotid artery and extending to the jugular bulb (Figure 3a, arrow), and a very small right-sided carotid body tumor. The vagal tumor was positive on octreotide and DOPA-PET scan. Both tumors showed slow growth on follow-up and have not been operated due to risks of morbidity. In the three years following diagnosis the patient experienced episodes of loss of consciousness, diagnosed as vasovagal syncope with an uncertain relationship to the vagal tumor, and an elevated 24hr urinary excretion of catecholamines without signs or symptoms of catecholamine excess. MRI of the abdomen revealed an 8mm nodule in the left adrenal, which was removed by adrenalectomy and described as an ‘adrenal medullary hyperplasia or pheochromocytoma’ on histopathologic examination.

Although the patient had several first and second-degree relatives with paraganglioma, and a known pathogenic mutation of *SDHD* (p.Asp92Tyr) in the maternal family, there was no known history of mutations or paraganglioma on the father’s side (Figure 3b). As the patient’s two younger siblings also carried the mutation, both underwent ENT and endocrinological investigations but no tumors or signs of catecholamine excess were found.

The analysis of genomic DNA from the patient and six other family members using microsatellite markers confirmed inheritance of a maternal chromosome containing a minimal haplotype defined by the D11S1793 (allele 133) and D11SS4090 (allele 183) alleles, present in all mutation carriers in the family (Figure 3c, yellow boxes). Although the father of the patient was not available for analysis, the paternal haplotype could be defined by alleles present in the patient and siblings but not present in any other family member. As both the patient and his two siblings share a paternal haplotype including the *SDHD* locus, a theoretical possibility exists that all three offspring inherited the mutation from the father. This would require the father to be a carrier of a mutation identical to that of the mother. However, as all three offspring also inherited a maternal mutation-bearing haplotype and homozygous mutation of *SDHD* is incompatible with life, possible genetic mechanisms allowing paternal inheritance are extremely improbable. The only likely explanation for these data, despite the lack of DNA from the father, is that the mutation was inherited from the mother in all three siblings.

The only tumor tissue available for analysis from this patient was an ‘adrenal medullary hyperplasia/pheochromocytoma’. Although the adrenal medulla showed clear signs of hyperplasia, this was insufficiently pronounced to allow a firm diagnosis. H&E sections showed a typical adrenal structure with an overprominent medulla (Figure 3d). Immunohistochemical staining with an anti-SDHB antibody showed clear loss of SDHB protein expression in the adrenal medulla (Figure 3dii). However, sequencing analysis of *SDHD* in normal and tumor samples provided no evidence for loss of the wild type (normal) allele in the tumor (Figure 3e), and microsatellite analysis showed no loss of chromosome 11 in the tumor (Figure 3f). This result could be due to admixture of normal cells with tumor cells, thereby masking loss of SDHD/chromosome 11 in tumors cells – a phenomenon common in paragangliomas. Another possibility, and one suggested by the profound loss of SDHB staining in the adrenal medulla, is that a non-genetic mechanism is mediating SDHD/SDHB loss in this tissue. While inconclusive in terms of SDHD p.Asp92Tyr-mediated pathogenicity, these data do show that this case of adrenal hyperplasia is SDH-related. However, speaking conservatively, we cannot definitively conclude that the patient’s p.Asp92Tyr mutation, a mutation proven by its dominant role in paraganglioma patients in the Netherlands and worldwide to be profoundly pathogenic, is the cause of the adrenal hyperplasia in this case. A firm conclusion will have to await the availability of new tumor tissue.

The third patient, a female aged 37, first presented with a feeling of fullness in the ears. A CT scan of the temporal bones revealed a 5 mm mass in the left middle ear and an enhancing lesion in the middle of the left ear cavity was visible on MRI, consistent with a jugulotympanic paraganglioma. Surgical resection and histological examination confirmed the diagnosis.

**Figure 4:**
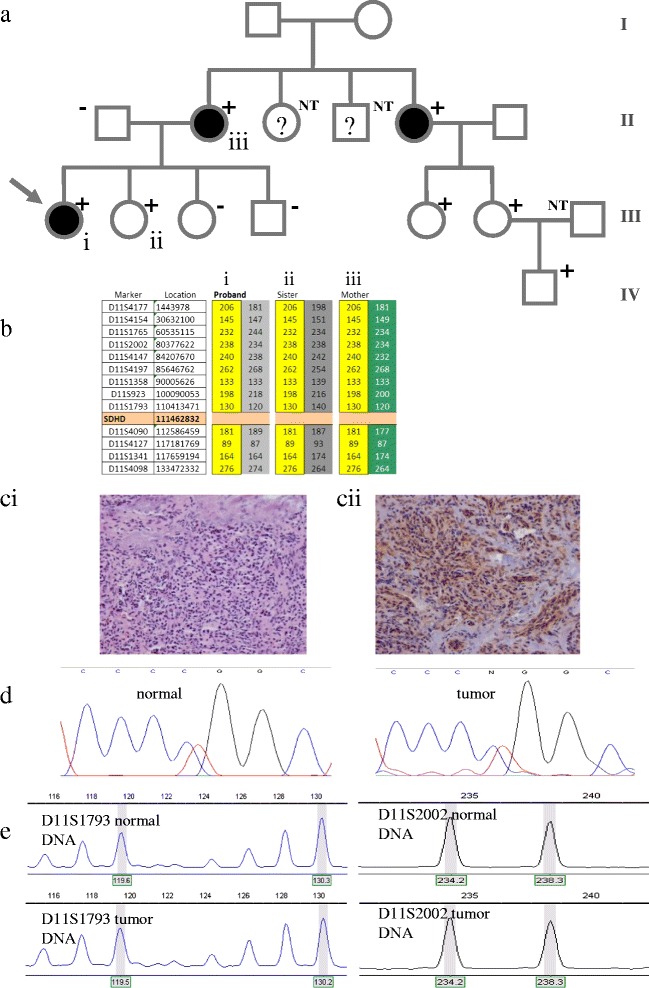
**Patient 4 – pedigree, DNA and tumor analysis. a)** Arrow indicates the proband, filled boxes indicate paraganglioma, plus or minus the *SDHD* mutation status, and question marks indicate suspicious but unconfirmed phenotypes. **b)** Chromosome 11 haplotypes of immediate family. Microsatellite markers are shown with genomic location. Alleles in yellow blocks represent the probable disease haplotype. **c)** Histochemistry and immunohistochemistry: c1) HE staining of JT PGL, 25×. c2) SDHB immunohistochemistry, 25×. **d)** Sanger sequencing of *SDHD* in normal (e1) and tumor (e2) DNA. **e)** Typical profiles of microsatellite marker alleles showing no loss of heterozygosity.

The patient had several maternal relatives with paragangliomas (Figure 4a) including the patient’s mother, who had a left carotid body paraganglioma removed at age 29 and was diagnosed with a right carotid body paraganglioma at age 58 (treated with radiation). The patient’s maternal aunt was diagnosed with a left jugulotympanic paraganglioma at age 34, and treated for recurrent disease at age 43. A maternal uncle, who died aged 38, was reportedly diagnosed with renal cancer at 29 years old, followed by occurrence of a tumor of the skull base at age 36, although neither diagnosis could be confirmed. All of the patient’s siblings (two sisters and one brother) were tumor-free and there were no other known diagnoses of paraganglioma in the family.

Based on the lack of known disease in the paternal family and a possible case of renal cancer, genetic testing of the *SDHB* gene was initially offered to the patient. This analysis failed to identify a mutation, but subsequent analysis of the *SDHD* gene resulted in the identification of a heterozygous mutation, c.284 T>C, p.Leu95Pro. Further testing in the family identified the mutation in the proband’s mother, one sister, two cousins (daughters of the patient’s maternal aunt with confirmed paraganglioma) and one of the cousin’s sons. Two additional cousins in the same sibship tested negative for the mutation. All remaining individuals in the family have not pursued genetic counseling to date.

Genomic DNA from three family members, all carriers of the p.Leu95Pro mutation, was available for microsatellite analysis and all three were found to share a common haplotype. Both daughters inherited a common haplotype from their mother. A scenario of paternal inheritance is effectively excluded in this patient by the differing paternal haplotypes carried by the patient and her sister, and by the common maternal haplotype (Figure 4b).

FFPE tumor tissue from patient 3 (Figure 3ci) showed normal staining for SDHB, indicating retention and normal expression of the protein (Figure 3cii). DNA sequencing analysis also showed retention of the wild type (normal) *SDHD* allele in the tumor (Figure 3d). Microsatellite analysis of tumor DNA from patient 3 (Figure 3e) showed an AIR of around 1.0, indicating that there was no loss of heterozygosity of chromosome 11. The normal expression of the SDHB protein and lack of evidence of genetic loss suggest that the p.Leu95Pro mutation, despite being a well-established pathogenic mutation, is not causative in this patient’s tumor.

In this study we present genetic and functional evidence supporting the *bone fide* maternal-related occurrence of a case (patient 1) of pheochromocytoma; a case with both an unequivocal clinical diagnosis and histological confirmation. We also present a case (patient 2) of head and neck paraganglioma with suggestive evidence for maternal-related tumorigenesis.

The ‘parent-of-origin’ related tumor phenotype of *SDHD* is one of the most unusual genetic manifestations in all of cancer biology and remains poorly understood. While the first recognition of this phenomenon immediately suggested the involvement of a maternally-imprinted gene [9], analysis of allele-specific expression in a variety of tissues has shown biallelic expression [4,17]. The complete constitutive silencing of one allele of *SDHD* is unlikely for several reasons, not least due to the specific loss of the maternal allele in *SDHD*-related paragangliomas, which is counterintuitive if one assumes that this allele is already completely inactivated by imprinting [18]. As patients with *SDHD*-related paraganglioma carry paternally-inherited mutations, a maternal imprint would result in a profound deficiency of succinate dehydrogenase activity, a situation known to cause major developmental defects even when residual enzyme activity is present [19]. Homozygous knockout of *Sdhd* in mice results in embryonic lethality [20,21].

A ‘parent-of-origin’ tumor phenotype is shared by a closely related gene, the recently identified succinate dehydrogenase assembly factor, SDHAF2. This gene encodes a protein involved in the addition of the flavin-adenine dinucleotide prosthetic group to form the active SDHA flavoprotein [7]. Although less central to SDH function than the *SDHB* and *SDHC* genes, both located on chromosome 1, *SDHAF2* shares one important characteristic with SDHD; both are located on chromosome 11. Chromosome 11 also harbors the main concentration of imprinted genes in the human genome, in the Ch11p15.5 region, with 11 genes expressed exclusively from the paternal or maternal chromosome and the opposite allele silenced by epigenetic mechanisms.

Functional loss of the maternal copy of chromosome 11 is a hallmark of *SDHD*-linked paragangliomas [18,22-24] and has led to the proposal that tumorigenesis occurs only when a paternally-transmitted mutation of *SDHD* and loss of the remaining non-imprinted maternal allele is accompanied by loss of a maternally-expressed imprinted modifier gene. Due to their common chromosomal location, *SDHD* (or *SDHAF2*) and an imprinted modifier can be targeted in a single genetic event involving whole chromosome loss. This ‘*SDHD*-imprinted modifier gene’ hypothesis has become known as the ‘Hensen Model’ [11,12,18]. Other studies have suggested that loss of the maternal copy of chromosome 11 may also be a factor in both sporadic pheochromocytomas and those related to mutations of the Von Hippel-Lindau (*VHL*) gene, located on chromosome 3 [25,26].

The ‘Hensen Model’ is relevant to the data presented here as it may explain the somatic recombination seen in the tumor of patient 1. This is an unusual event in itself and has only previously been described in one case of paraganglioma [13]. Hensen and colleagues (2004) predicted exactly the scenario now observed; “When the *SDHD* mutation is maternally-transmitted, at least two events caused by different chromosomal mechanisms will be required… namely loss of the paternal wild-type *SDHD* allele by, for example, mitotic recombination, followed by loss of the recombined paternal chromosome containing the paternal 11q23 region and the maternal 11p15 region”.

The third patient represents the first reported case of a phenocopy in PGL. A phenocopy in a family with a hereditary tumor burden is an individual with a tumor that is unrelated to the familial genotype, a frequent phenomenon in families with common tumors such as those of the breast or colon. This case also serves to demonstrate that causality cannot be taken for granted, even in patients with well-described pathogenic mutations. To rule out misdiagnosis of the tumor, morphology and immunohistochemical characteristics were closely scrutinized both initially, and subsequent to the evaluation of the genetic and SDHB immunohistochemical analysis results. Two independent pathologists confirmed the diagnosis of jugulotympanic paraganglioma, and all morphological and histochemical indicators supported this conclusion.

## Conclusions

At least two cases of maternally-inherited *SDHD*-related tumor susceptibility have now been described (this study and [13]). This presents genetic counselors with a challenge, as it is now no longer possible to unequivocally state that a carrier of a maternally-transmitted mutation will never develop a tumor. Despite this development, we consider the increase in risk represented by these reports to be negligible. Carriers of maternally-inherited *SDHD* mutations can still be assured that their lifetime risk is not significantly different to non-mutation carriers. Whether this report will stimulate clinicians to re-evaluate carriers of maternally-inherited mutations, leading to the recognition of further cases, remains to be seen.
